# Supplementary material for: Use of allele-specific qPCR and PCR-RFLP analysis for rapid detection of the SARS-CoV-2 variants in Tunisia: A cheap flexible approach adapted for developing countries
Source: PLoS One. 2025 May 5;20(5):e0321581. doi: 10.1371/journal.pone.0321581 (PMC12052121; doi:10.1371/journal.pone.0321581)
Supplement: S3 Table — (DOCX) [file pone.0321581.s003.docx]

|  | Targets  (gene) | Recombinant plasmid copy numbers | | | | | | | |
| --- | --- | --- | --- | --- | --- | --- | --- | --- | --- |
|  |  | 5x10^7^ | 5x10^6^ | 5x10^5^ | 5x10^4^ | 5x10^3^ | 5x10^2^ | 5x10^1^ | 5x10^0^ |
| Intra-assay | ΔHV69-70  (S) | 1.87 | 1.60 | 0.41 | 0.38 | 0.41 | 0.53 | 0.32 | 2.25 |
|  | HV69-70  (S) | 1.29 | 0.96 | 1.16 | 1.07 | 0.19 | 0.19 | 0.45 | 0.17 |
|  | ΔKSF141-143 (ORF1a) | 0.85 | 1.15 | 0.55 | 0.05 | 0.35 | 0.65 | 0.45 | - |
|  | KSF141-143 (ORF1a) | 1.52 | 0.88 | 0.25 | 0.48 | 0.73 | 0.84 | 0.13 | - |
| Inter-assay | ΔHV69-70  (S) | 1.29 | 1.18 | 0.46 | 0.76 | 1.20 | 0.57 | 0.30 | 2.59 |
|  | HV69-70  (S) | 1.10 | 1.45 | 1.86 | 2.03 | 1.43 | 0.57 | 1.04 | 1.41 |
|  | ΔKSF141-143 (ORF1a) | 1.84 | 2.12 | 0.46 | 0.47 | 0.50 | 1.26 | 0.51 | - |
|  | KSF141-143 (ORF1a) | 1.15 | 2.06 | 1.70 | 0.78 | 1.07 | 1.45 | 3.63 | - |

|  |
| --- |
