# Supplementary figures and images for: Use of allele-specific qPCR and PCR-RFLP analysis for rapid detection of the SARS-CoV-2 variants in Tunisia: A cheap flexible approach adapted for developing countries
Source: PLoS One. 2025 May 5;20(5):e0321581. doi: 10.1371/journal.pone.0321581 (PMC12052121; doi:10.1371/journal.pone.0321581)

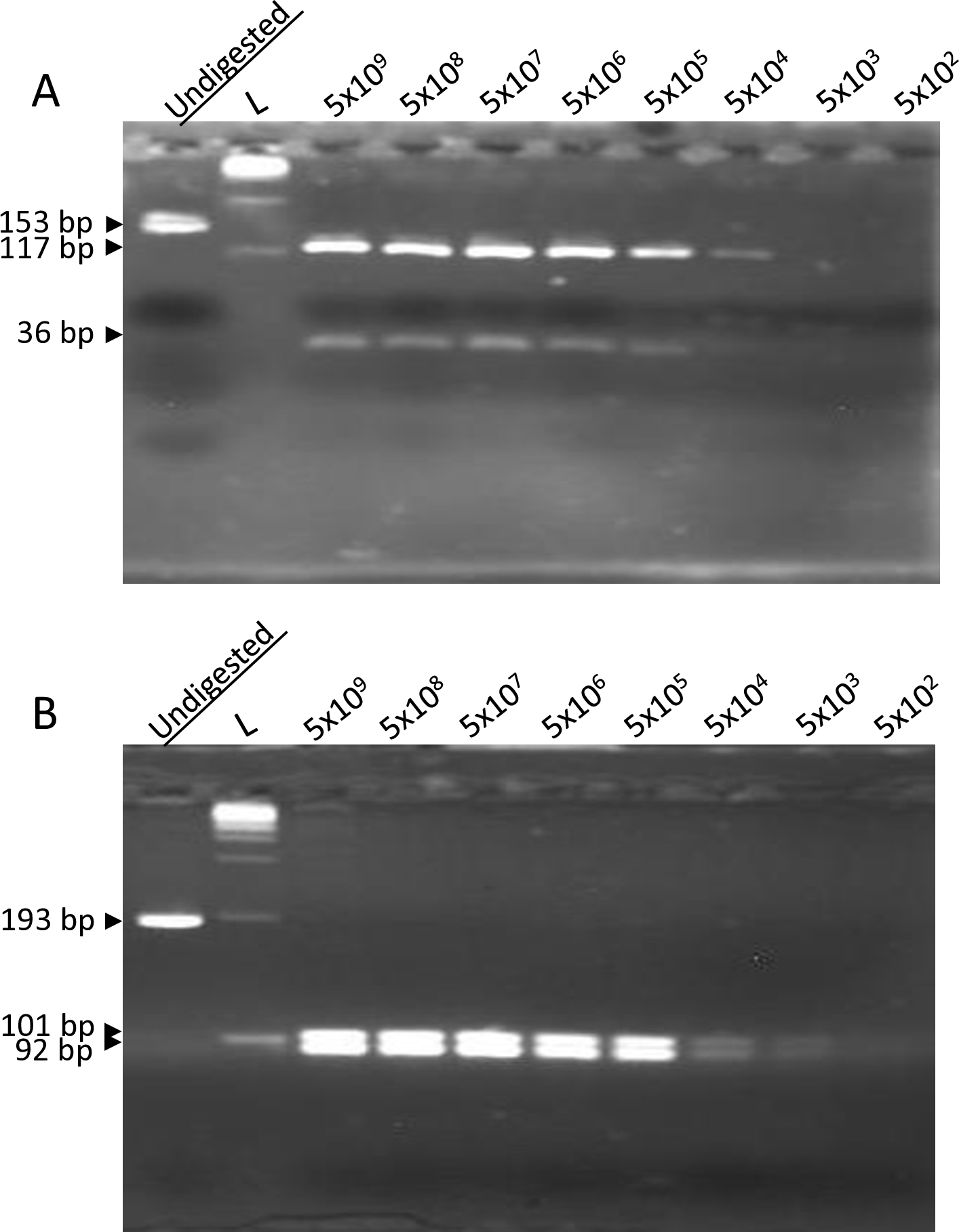

Supplement: S1 Fig — A: PCR amplification and NlaIII digestion of OFR1b gene (aa 254–303) detecting the Y264H mutation. B: PCR amplification and BfaI digestion of S gene (aa 412–475) detecting the two successive mutations V445P and G446S. L: 100 bp DNA ladder. (TIF) [file pone.0321581.s004.tif]
