# Supplementary material for: Use of allele-specific qPCR and PCR-RFLP analysis for rapid detection of the SARS-CoV-2 variants in Tunisia: A cheap flexible approach adapted for developing countries
Source: PLoS One. 2025 May 5;20(5):e0321581. doi: 10.1371/journal.pone.0321581 (PMC12052121; doi:10.1371/journal.pone.0321581)
Supplement: S1 Raw image — (PDF) [file pone.0321581.s005.pdf]

Original gel image for Fig. 3A.

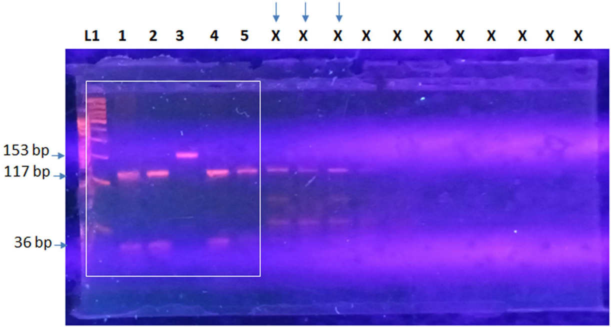

PCR amplification and NlaIII digestion of OFR1b gene (aa 254-303) detecting the Y264H mutation characterizing BQ.1 sublineages in lanes 1, 2, 4 and 5. Lane L1: 50 bp DNA ladder. X: the lane not included in the final figure. In the first three lanes X, where samples with low amounts of cDNA were used, non-specific amplification and/or primer-dimer formation (seizes between 50 and 100 bp) were observed. These issues were particularly noticeable when primers were used at a concentration of 0.3 µM. However, this problem was resolved by reducing the primer concentration to 0.15 µM, as showed in the original gel image for S1 Fig (A). DNA fragments were resolved on 8% polyacrylamide gel and stained with ethidium bromide.

Original gel image for Fig. 3B.

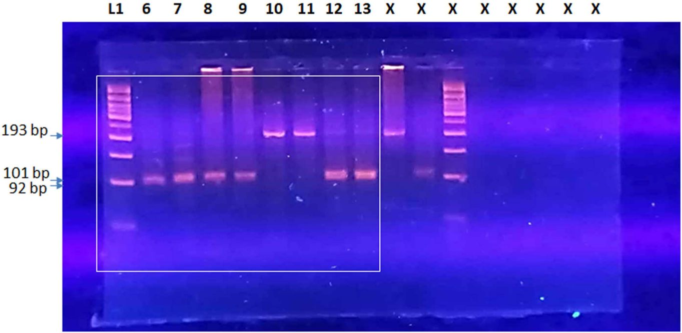

PCR amplification and BfaI digestion of S gene (aa 412-475) detecting the two successive mutations V445P and G446S characterizing XBB sublineages in lanes 6, 7, 8, 9, 12 and 13. Lane L1: 50 bp DNA ladder. X: the lane not included in the final figure. DNA fragments were resolved on 8% polyacrylamide gel and stained with ethidium bromide.

Original gel image for Fig. 3C.

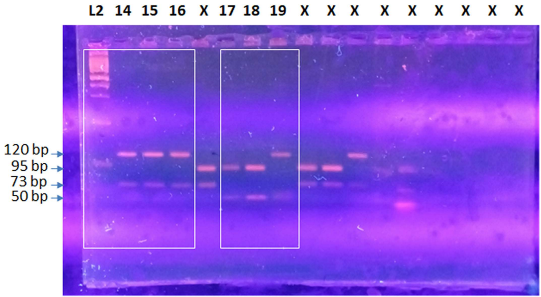

PCR amplification and MspI/DdeI digestion of S gene (aa 412-475) detecting the two mutations K444X (T, R, N or M) and L452Q of the BA.5 descendent sublineages in lanes 14, 15 and 16; the N460K (AAG) mutation characterizing the parental BA.2.75 sublineage in lanes 17 and 18; the three mutations K444X (T, R, N or M), L452Q and N460K (AAG) characterizing CH1.1. sublineage in lane 19. Lane L2: 100 bp DNA ladder. X: the lane not included in the final figure. DNA fragments were resolved on 8% polyacrylamide gel and stained with ethidium bromide.

Original gel image for S1 Fig (A).

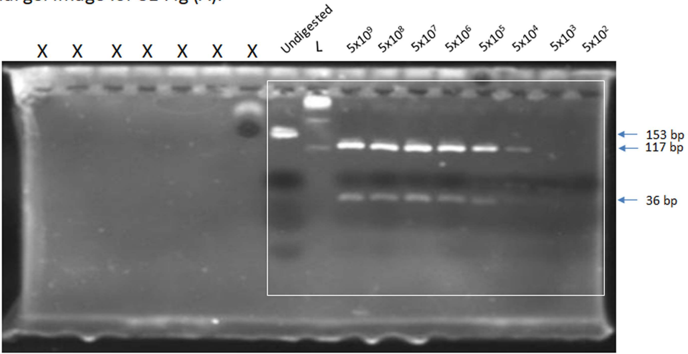

PCR amplification and NlaIII digestion of OFR1b gene (aa 254-303) detecting the Y264H mutation using ten-fold serial dilutions of recombinant plasmid DNA ranging from 5×10^9 to 5×10^2 copies per reaction. Lane L: 100 bp DNA ladder. X: the lane not included in the final figure. DNA fragments were resolved on 8% polyacrylamide gel and stained with ethidium bromide.

Original gel image for S1 Fig (B).

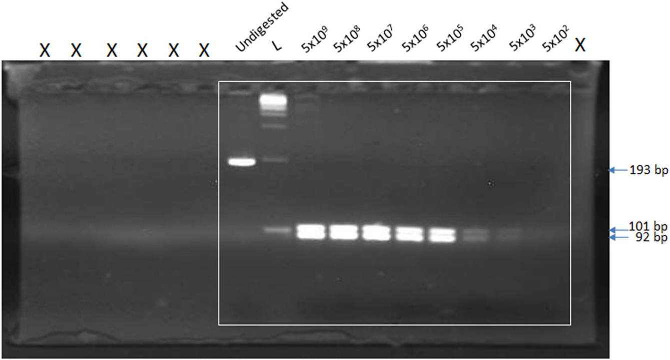

PCR amplification and BfaI digestion of S gene (aa 412-475) detecting the two successive mutations V445P and G446S using ten-fold serial dilutions of recombinant plasmid DNA ranging from 5×10^9 to 5×10^2 copies per reaction. L: 100 bp DNA ladder. X: the lane not included in the final figure. DNA fragments were resolved on 8% polyacrylamide gel and stained with ethidium bromide.
